# Kinetic Analysis of SARS-CoV-2 S1–Integrin Binding Using Live-Cell, Label-Free Optical Biosensing

**DOI:** 10.3390/bios15080534

**Published:** 2025-08-14

**Authors:** Nicolett Kanyo, Krisztina Borbely, Beatrix Peter, Kinga Dora Kovacs, Anna Balogh, Beatrix Magyaródi, Sandor Kurunczi, Inna Szekacs, Robert Horvath

**Affiliations:** 1 Nanobiosensorics Laboratory, Institute of Technical Physics and Materials Science, HUN-REN Centre for Energy Research, Konkoly-Thege Miklós út 29-33, H-1121 Budapest, Hungary; kanyo.nicolett@ek.hun-ren.hu (N.K.); borbely.krisztina@ek.hun-ren.hu (K.B.); peter.beatrix@ek.hun-ren.hu (B.P.); kovacs.kinga.dora@ek.hun-ren.hu (K.D.K.); balogh.anna@ek.hun-ren.hu (A.B.); magyarodi.beatrix@ek.hun-ren.hu (B.M.); kurunczi.sandor@ek.hun-ren.hu (S.K.); inna.szekacs@ek.hun-ren.hu (I.S.); 2 Chemical Engineering and Material Science Doctoral School, University of Pannonia, Egyetem u.10, H-8200 Veszprém, Hungary; 3 Department of Biological Physics, Eötvös University, Pázmány Péter Sétny. 1/C, H-1117 Budapest, Hungary; 4 Institute of Biophysics, HUN-REN Biological Research Centre, H-6726 Szeged, Hungary

**Keywords:** SARS-CoV-2 spike protein, integrin binding, RGD motif, label-free optical biosensing, resonant waveguide grating, HeLa cell adhesion

## Abstract

The SARS-CoV-2 spike (S1) protein facilitates viral entry through binding to angiotensin-converting enzyme 2 (ACE2), but it also contains an Arg–Gly–Asp (RGD) motif that may enable interactions with RGD-binding integrins on ACE2-negative cells. Here, we provide quantitative evidence for this alternative binding pathway using a live-cell, label-free resonant waveguide grating (RWG) biosensor. RWG technology allowed us to monitor real-time adhesion kinetics of live cells to RGD-displaying substrates, as well as cell adhesion to S1-coated surfaces. To characterize the strength of the integrin–S1 interaction, we determined the dissociation constant using two complementary approaches. First, we performed a live-cell competitive binding assay on RGD-displaying surfaces, where varying concentrations of soluble S1 were added to cell suspensions. Second, we recorded the adhesion kinetics of cells on S1-coated surfaces and fitted the data using a kinetic model based on coupled ordinary differential equations. By comparing the results from both methods, we estimate that approximately 33% of the S1 molecules immobilized on the Nb_2_O_5_ biosensor surface are capable of initiating integrin-mediated adhesion. These findings support the existence of an alternative integrin-dependent entry route for SARS-CoV-2 and highlight the effectiveness of label-free RWG biosensing for quantitatively probing virus–host interactions under physiologically relevant conditions without the need of the isolation of the interaction partners from the cells.

## 1. Introduction

The entry of severe acute respiratory syndrome coronavirus 2 (SARS-CoV-2) into host cells is primarily mediated by the angiotensin-converting enzyme 2 (ACE2), which binds to the receptor-binding domain (RBD) of the viral spike (S1) protein and initiates viral fusion and replication [1,2,3]. However, the complexity of virus–host interactions extends beyond this canonical pathway. Recent studies suggest that other membrane-associated structures, such as glycocalyx components, proteoglycans, and integrins, also contribute to viral attachment, internalization, and tissue tropism [4,5]. These auxiliary factors may help explain the broad cellular tropism of SARS-CoV-2 and its ability to infect ACE2-low or -negative tissues.

Among these alternative entry factors, integrins are particularly interesting due to their ability to recognize Arg–Gly–Asp (RGD) motifs found in extracellular matrix (ECM) proteins. Integrins are heterodimeric transmembrane receptors composed of α and β subunits that mediate cell–ECM adhesion and bidirectional intracellular signaling [6]. Several viruses, such as adenoviruses and human cytomegalovirus, exploit integrins for cell entry, using RGD or RGD-like sequences to initiate receptor-mediated endocytosis or to trigger intracellular signaling cascades that promote infection [7].

The S1 subunit of the SARS-CoV-2 spike protein contains an RGD-like sequence located near the RBD, suggesting that integrins may serve as co-receptors or even independent entry points into host cells [7]. This interaction is particularly relevant in cell types that do not express ACE2 or in tissues where ACE2 is present at low levels [8]. In vitro studies have reported specific binding between recombinant S1 or S1-RBD proteins and isolated integrins such as αvβ3, α5β1, and αvβ6 [9,10,11]. These findings support the hypothesis that integrins could play a key role in SARS-CoV-2 pathogenesis, either by enhancing viral entry or by modulating downstream signaling pathways associated with inflammation and immune evasion [12]. Norris et al. (2023) reported weak binding between the SARS-CoV-2 S1-RBD and αvβ3 integrin (dissociation constant of S1, KdS13D > 500 nM), and moderate affinity for αvβ6 (KdS13D = 230 ± 180 nM), both measured in a purified, non-cellular surface plasmon resonance (SPR) system without membrane context [11]. In contrast, Bugatti et al. (2022) observed a markedly stronger interaction between recombinant SARS-CoV-2 RBD and αvβ3 integrin in a similar SPR setup, reporting a kinetic KdS13D of 6.3 nM and a steady-state KdS1 3D of 59.3 nM [10].

Notably, Liu et al., (2022) demonstrated that while recombinant S1 protein binds strongly to purified α5β1 integrin—with a KdS1 3D of approximdately 31 nM as determined by SPR—binding was weaker when S1 was applied to live cells expressing surface integrins in flow cytometry and internalization assays [9]. Although no specific KdS1 3D values were reported for the cell-based experiments, the authors attributed the reduced binding to limitations associated with membrane-bound integrins, such as restricted accessibility, variable orientation, and activation state [9]. This highlights the importance of studying integrin–S1 interactions in both purified and cellular contexts to understand their relevance in vivo. The reported KdS1 3D values from these studies vary depending on the viral ligand used and the experimental method applied; these values are summarized in Table 1.

Given their roles in cell adhesion, signaling, and viral entry, integrins have emerged as promising therapeutic targets. Integrin antagonists—including RGD mimetic compounds, small molecules, or monoclonal antibodies—may block these auxiliary pathways, and ongoing research is evaluating whether combining ACE2-directed therapies with integrin inhibitors can synergistically prevent SARS-CoV-2 entry and mitigate inflammatory cascades associated with severe coronavirus disease 2019 (COVID-19) [13].

To address these limitations, cell-based biosensing platforms offer a promising alternative. Optical biosensor techniques have emerged as promising and popular tools for SARS-CoV-2 spike protein detection due to their significant advantages, such as affordability, high sensitivity, and simple operation [14]. Live-cell, label-free optical biosensors—such as those based on resonant waveguide grating (RWG) technology—allow real-time tracking of cellular adhesion to functionalized surfaces. This approach preserves the membrane architecture and receptor dynamics while enabling quantitative analysis of ligand–receptor interactions under physiologically relevant conditions [2,15,16]. In particular, HeLa cells provide an excellent model system for studying ACE2-independent viral entry, as they express multiple RGD-binding integrins—including α5β1, αvβ3, αvβ5, and αvβ6—but lack endogenous ACE2 expression [17,18].

In this study, we applied a high-throughput RWG live-cell optical biosensor that enables real-time, quantitative monitoring of cell adhesion kinetics via integrin–ligand interactions in the native membrane environment. We investigated whether the full-length SARS-CoV-2 S1 subunit can directly mediate adhesion of HeLa cells via integrin binding and compared our results to previously reported S1–integrin affinity values (see Table 1). To validate the specificity of this interaction, we also performed inhibition assays using soluble S1 protein to test whether integrin–extracellular matrix (ECM) binding can be competitively blocked. The dissociation constant (KdS1 3D) was calculated using two complementary approaches: based on live-cell adhesion kinetics to surface-immobilized S1 and from the dose-dependent inhibition curves obtained with soluble S1.

The aim of this work was to provide quantitative, cell-based evidence for integrin engagement by the SARS-CoV-2 S1 subunit, independent of ACE2, and to characterize the molecular and spatial parameters that govern this interaction. By leveraging a label-free biosensor platform and kinetic modeling, we sought to expand our understanding of alternative SARS-CoV-2 entry mechanisms and to support the development of antiviral strategies targeting integrin–virus interactions.

## 2. Materials and Methods

### 2.1. Cell Culture and Sample Preparation

HeLa cells (ECACC 93021013) were maintained in Dulbecco’s modified Eagle’s medium (DMEM) supplemented with 10% fetal bovine serum (FBS), 4 mM L-glutamine, and 100 U/mL penicillin and 100 μg/mL streptomycin solution. Cells were cultured in a humidified incubator at 37 °C with 5% CO_2_. Before biosensor measurements, cells were harvested by washing with phosphate-buffered saline (PBS) and treated with 1 mL trypsin-EDTA for 2 min to detach them from the culture surface. The trypsinization reaction was stopped by adding 1 mL DMEM, and the cells were centrifuged at 200× *g* for 5 min. The supernatant was removed, and the pellet was resuspended in 1 mL of assay buffer (HEPES-HBSS, 20 mM 2-[4-(2-hydroxyethyl)piperazin-1-yl]ethanesulfonic acid (HEPES) in Hank’s balanced salt solution (HBSS), pH 7.4). After a second centrifugation, the supernatant was discarded, and the cells were resuspended in 1 mL of assay buffer for subsequent measurements.

### 2.2. S1 Coating Measurement

To assess the adhesion of HeLa cells to SARS-CoV-2 S1 protein via integrin-mediated interactions, the biosensor surface was coated with recombinant S1 protein. A stock solution of S1 protein (6 µL) was diluted in 30 µL of PBS, and 12 µL of this solution was added to three separate wells of the biosensor plate. The plate was incubated at room temperature for 60 min to allow protein adsorption onto the surface. After incubation, the wells were washed twice with 50 µL of 10 mM HEPES to remove unbound S1 protein. A baseline measurement was recorded in 25 µL of 10 mM HEPES to quantify any remaining unbound S1 protein.

To minimize nonspecific interactions, the surface was blocked using PP (poly(L-lysine)-graft-poly(ethylene glycol) [PLL(20)-g(3.5)-PEG(2)]) (SuSoS AG, Dübendorf, Switzerland)). The HEPES was removed, and 25 µL of PP (0.5 mg/mL) was added to each well. The plate was incubated for 30 min at room temperature. Following incubation, the PP solution was removed, and the wells were washed twice with 50 µL of 20 mM HEPES HBSS to eliminate excess blocking reagent. A second baseline measurement was taken using 25 µL HEPES-HBSS for 30 min to establish the final background signal before cell addition.

**Figure 1:**
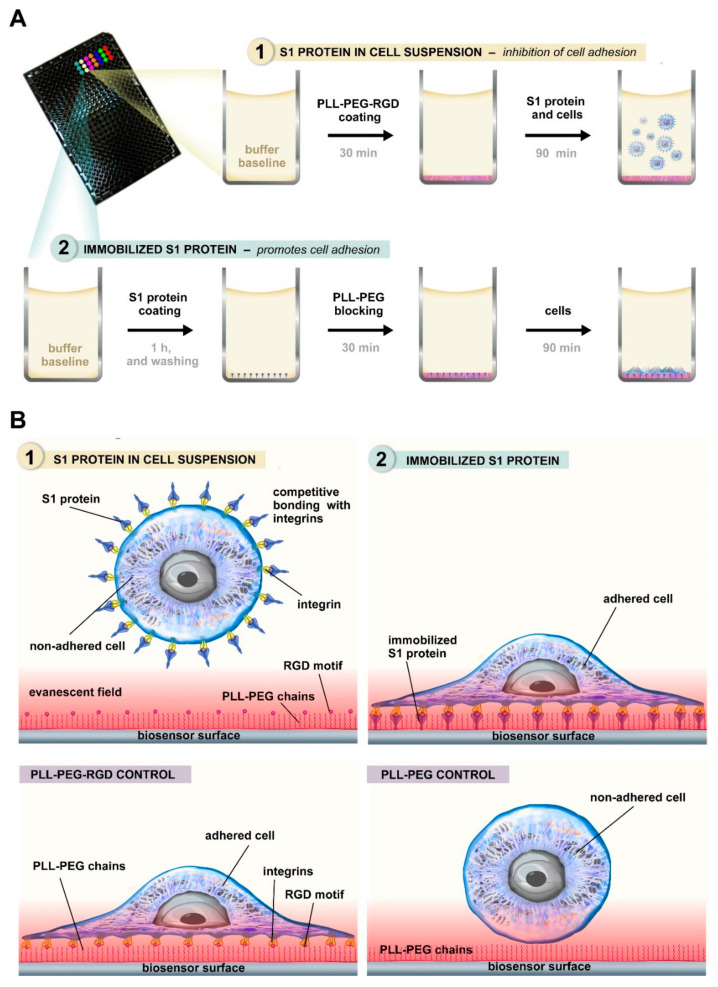
Experimental scheme and molecular mechanism of integrin-mediated adhesion assays. (**A**) Experimental workflow of the two biosensor assay setups: (1) inhibitory effect of soluble S1 protein in cell suspension and (2) adhesion to surface-immobilized S1 protein. (**B**) Schematic representation of molecular interactions during the assays. Soluble S1 protein blocks integrins via competitive binding, preventing RGD-mediated adhesion (top left), while immobilized S1 protein supports adhesion through integrin engagement (top right). PPR surface promotes integrin-mediated adhesion (bottom left), whereas PP alone prevents it (bottom right).

For biosensor measurements, HeLa cells were diluted to 8000 cells per well, and 25 µL of the cell suspension was added to each well of the sensor plate. Real-time adhesion was monitored for 1.5 h using the label-free biosensor system, allowing quantitative assessment of HeLa cell binding to the S1-coated surface. The wavelength shift was recorded over time to evaluate the extent of cell adhesion and determine the role of integrin-mediated interactions in S1 protein binding (Figure 1).

### 2.3. S1 Protein Inhibition Assay

To evaluate whether soluble SARS-CoV-2 S1 protein inhibits HeLa cell adhesion to PPR (PLL-g-PEG-DBCO-Mal)-CKK-(Acp)-(Acp)-(Acp)-GRGDS-coated (SuSoS AG, Dübendorf, Switzerland) surfaces via integrin receptor blocking, cells were pre-incubated with S1 protein in suspension prior to biosensor measurements. Biosensor wells were first coated with 50% PPR (PP and PPR in 1:1 ratio) (0.5 mg/mL) in 30 µL per well and incubated for 30 min at room temperature to allow surface adsorption. Following incubation, the wells were washed with 25 µL of 20 mM HEPES-HBSS, and excess liquid was removed.

To assess the effect of soluble S1 protein on cell adhesion, HeLa cells (8000) were incubated with either S1 protein or BSA as a control. The final concentration of S1 protein and BSA in the wells with HeLa cells were 2.67 × 10^−1^ µM. BSA served as a non-specific protein control. The final mixtures were distributed across three wells, with 25 µL of cell suspension per well (Figure 1). BSA was not used as a surface blocker but as a soluble competitor to assess whether non-specific protein binding could inhibit integrin-mediated adhesion. Given its molecular weight (66 kDa) and widespread use in adsorption and competition assays, BSA serves as a standard control protein. The lack of inhibitory effect observed with BSA (unlike soluble S1) confirms that the observed reduction in adhesion is specific to S1–integrin interactions.

Real-time adhesion was monitored using the label-free biosensor system for 90 min. The resulting wavelength shift was recorded over time to determine the extent of cell adhesion. The effect of soluble S1 protein was compared to control conditions to evaluate whether pre-incubation with S1 protein reduced integrin-mediated binding to the PPR-coated surface.

### 2.4. Basic Sensing Mechanism of the Epic BT RWG Biosensor System

The RWG-based label-free optical biosensor approach, offering sub-picometer resolution and a surface mass sensitivity of approximately 3 pg/mm^2^ [2], enables quantitative kinetic analysis of cell adhesion processes. This technique detects changes in the local refractive index near the sensor surface, which are reported as a wavelength shift (∆λ) of the resonant peak. The magnitude of ∆λ correlates directly with the amount of mass (e.g., cells, proteins) accumulating on or interacting with the sensor surface. Its compatibility with 96- or 384-well SBS-format microplates also allows high-throughput, parallel measurements in living cells. Although the Corning Epic system is no longer commercially available, we used a fully functional Epic BT device with pre-stocked microplates for all experiments. The bottom of the Epic microplate is covered with a thin, high-refractive index, biocompatible layer of niobium pentoxide, and each well contains a 2 × 2 mm^2^ optical grating that functions as a biosensor. When illuminated at the resonant wavelength, these form a so-called evanescent electromagnetic field with a penetration depth of 150 nm, which decays exponentially with distance from the surface. Changes in the refractive index in the evanescent field can be induced by various events, including molecular adsorption or cell mass rearrangement, which cause a shift in the resonant wavelength. In each microplate well, the resulting resonant wavelength shift can be detected in real time with a time resolution of 3 s [15,19,20].

### 2.5. Assessment of S1 Surface-Density-Dependent Adhesion

To probe how the density of adsorbed S1 protein influences HeLa cell attachment, we first prepared a series of surface coatings from our 1.6 µM (0.2 mg/mL) S1 stock by diluting in PBS to final concentrations of (2.67 × 10^−1^; 5.33 × 10^−2^; 1.06 × 10^−2^; 2.13 × 10^−3^; 4.27 × 10^−4^ and 8.53 × 10^−5^ µM). A total of 12 µL of each dilution was added to individual wells of the biosensor plate and incubated at room temperature for 60 min to allow protein adsorption. After two washes with 50 µL of 10 mM HEPES to remove unbound S1, a baseline signal was recorded in 10 mM HEPES. Non-specific sites were then blocked by adding 25 µL of 0.5 mg/mL PP and incubating for 30 min, followed by two washes with 50 µL of HEPES-HBSS and a second baseline measurement in 25 µL of 20 mM HEPES-HBSS to establish the final background.

HeLa cells were seeded at 8000 cells per well (25 µL) and real-time adhesion was monitored for 90 min. By comparing the wavelength-shift traces across the six S1 densities, we identified the minimum surface concentration at which integrin-mediated adhesion reached saturation.

### 2.6. Statistics and Kinetic Analysis

All experiments were performed at least in minimum three parallel measurements (*n* ≥ 3). Data are presented as mean ± standard deviation (SD). Curve fitting for affinity determinations was carried out using nonlinear regression in Python 3.12.3.

To quantitatively model ligand-receptor binding dynamics on cell surfaces, we implemented a fitting pipeline in Python using the open-source SciPy library. The kinetic adhesion model was defined as a system of coupled ordinary differential equations described by Kanyo et al. [15], which we numerically solved using the scipy.integrate.odeint function. We employed scipy.optimize.curve_fit for parameter estimation, which applies a nonlinear least squares algorithm to minimize the difference between model predictions and experimental data.

While this approach relies on simplifying assumptions (e.g., average cell area, effective receptor number), similar kinetic modeling frameworks have been used in other live-cell systems such as other adhesion kinetics studies in immunology and neurobiology [21,22]. These methods provide physiologically relevant insights into binding strength even in the absence of exact receptor quantification.

Given the inherent stiffness of the ODE system—where small numerical inaccuracies can significantly influence solutions—we observed strong sensitivity to initial parameter guesses. This sensitivity stems from the complex structure of the cost function, which may contain multiple local minima. As a result, the fitting outcome often varied with different initial conditions, and a single optimization run could not reliably identify globally optimal parameters.

To address this challenge, we conducted multiple fits for each dataset by systematically varying the initial parameter values across a predefined grid. The parameter set that yielded the highest coefficient of determination (R^2^) was selected as the best fit. This process allowed us to robustly estimate the parameters governing the dynamics of the system. Final parameter values reported in the results were derived from averaging the best-fit parameters across replicate measurements. All visualizations, including model fits, parameter distributions, and dynamic behavior over time, were generated using Matplotlib (version 3.9.0).

## 3. Results and Discussion

**Figure 2:**
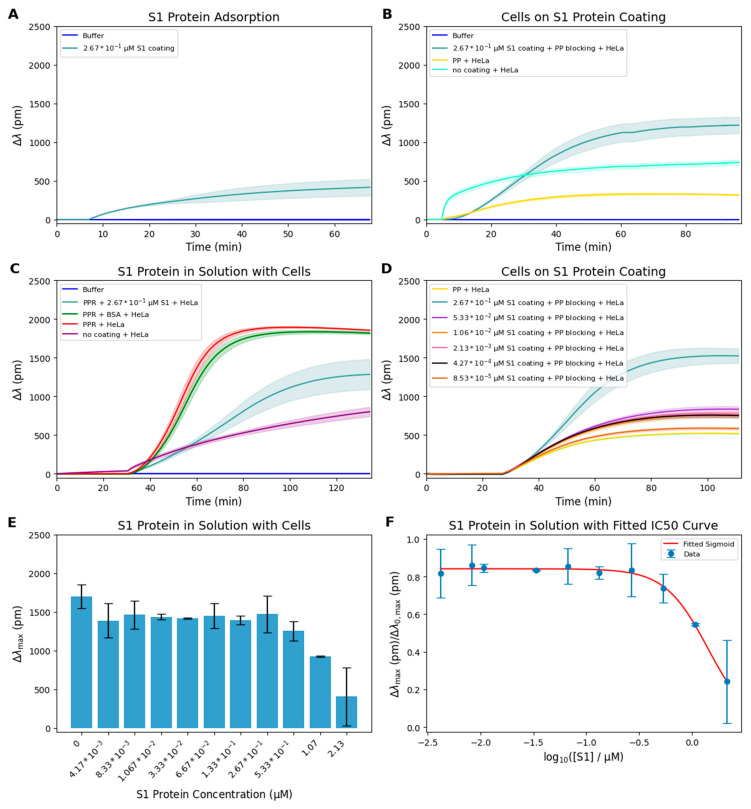
HeLa cell adhesion to SARS-CoV-2 S1 protein-coated or RGD-displaying biosensor surfaces measured by RWG. (**A**) S1 protein adsorption onto the surface at 0.267 µM, with buffer control (*n* = 3). (**B**) Effect of PP blocking on the S1-coated surface–HeLa cell adhesion to S1-coated surfaces (0.267 µM) with PP blocking, compared to uncoated and PP-only surfaces (*n* = 3). (**C**) HeLa cell adhesion to PPR-coated surfaces in the presence of soluble S1 protein (0.267 µM), with appropriate controls (*n* = 3). (**D**) Cell adhesion to surfaces coated with different concentrations of S1 protein (0.267–0.085 µM) (*n* = 3). (**E**) Quantification of Δλ_max_ values in response to soluble S1 protein added to HeLa cells on PPR-coated surfaces (*n* = 3). (**F**) Normalized adhesion response calculated from panel E and plotted against the log concentration of S1, with a fitted sigmoidal IC_50_ curve.

To assess the efficiency of S1 immobilization and confirm that HeLa cell adhesion is driven specifically by RGD-recognizing integrins, we performed real-time RWG binding and blocking assays (Figure 2).

In the first experiment, we investigated the interaction between the SARS-CoV-2 S1 protein and the bare waveguide surface. We found that stable and measurable protein adsorption occurred, as indicated by the increasing wavelength shift in S1 protein film (Δλ_S1_) recorded by the optical biosensor (Figure 1A).

Since the calibration of the biosensor signal to surface mass density was obtained using polyelectrolyte multilayers a correction has to be employed when calculating protein surface mass densities. To quantify the actual amount of SARS-CoV-2 S1 protein bound to the sensor surface in mass units, the corrected surface mass density (Γ_corrected_) was obtained by multiplying the measured wavelength shift (Δλ) by a sensitivity factor of 0.31 ng/cm^2^/pm, where surface mass densities determined from optical waveguide lightmode spectroscopy measurements were correlated with responses measured using an RWG imager. The resulting linear relationship between Δλ and surface mass (ΔM) provided the calibration slope of 0.31 ng/cm^2^/pm, which was used throughout our analysis, based on data reported by Orgovan et al., 2014 [23].

To account for the differences in optical properties between proteins and polyelectrolytes, we applied an additional correction. The refractive index increment (dn/dc) was taken as 0.1850 cm^3^/g for the S1 protein [24], and 0.1955 cm^3^/g for the polyelectrolyte layer (based on the average of poly(allylamine hydrochloride) (PAH) and poly(sodium 4-styrenesulfonate) (PSS) [25]. Accordingly, the measured Δλ_S1_ value (354 pm) was corrected using the following equation:

(1) $${\;\Gamma }_{corrected}={\Delta \lambda }_{S1}\;\times \frac{\frac{dn}{dc}\;polyelectrolyte}{\frac{dn}{dc}\;protein}\;\times \;0.31\frac{\frac{ng}{{cm}^{2}}}{pm}=115.97\frac{ng}{{cm}^{2}}$$

Thereafter, the molar surface density ($\rho_{RGD}$) was then calculated based on the molecular weight ($M_{S1}$) of the S1 protein (125 kDa):

(2) $$\rho_{RGD}=\frac{{\;\Gamma }_{corrected}}{M_{S1}}=\frac{115.97}{125\;000}\frac{\frac{ng}{{cm}^{2}}}{\frac{ng}{nmol}}=9.28\times 10^{-4}\frac{nmol}{{cm}^{2}}$$

The molar surface density was then used to calculate the surface density of RGD motifs (ν_RGD_, molecules/µm^2^) by multiplying σ by Avogadro’s number (Nₐ = 6.022 × 10^23^ mol^−1^). This yielded a surface density of 5568 molecules/µm^2^ for the concentrated S1 solution, which was achieved using a 2.67 × 10^−1^ (µM) S1 solution. The molar surface density was then used to calculate the surface density of RGD motifs (ν_RGD_, molecules/µm^2^) by multiplying σ by Avogadro’s number (Nₐ = 6.022 × 10^23^ mol^−1^). This yielded a surface density of 5568 molecules/µm^2^ for the concentrated S1 solution, which was achieved using a 1.6 µM S1 stock solution. It is important to clarify that only approximately 33% of the immobilized S1 protein is functionally active and capable of interacting with cellular integrins under our experimental conditions. This value was estimated based on the fitted KdS1 3D (1.116 µM) obtained from competitive inhibition assays using soluble S1 protein and is further supported by simulations presented in Supplementary Figure S3. Although the kinetic fitting model assumes full activity of surface-bound S1 by default, this assumption clearly overestimates the number of available binding sites when not all immobilized molecules are available for integrin binding. The derived dissociation constants should therefore be interpreted as effective values that inherently reflect this limited active fraction. According to ELISA results provided by the manufacturer (Abcam) for the recombinant SARS-CoV-2 S1 protein (ab273068), the adsorbed protein retains antigen-binding functionality, but our data indicate that only a subset of these molecules adopt an integrin-accessible conformation after immobilization [26]. To assess surface properties, we performed contact angle measurements on the Nb_2_O_5_-coated biosensor, revealing intermediate wettability (61.8–76.3°), which reduces the risk of protein denaturation (Figure S4). The calculated surface mass density (116 ng/cm^2^) and the plateau in the adsorption curve confirm that a single monolayer of S1 is formed. Multilayer formation is excluded based on protein spacing and functional adhesion. Additionally, HeLa adhesion experiments, including PP blocking and competitive inhibition with soluble S1, support the conclusion that a sufficient number of integrin-recognizable RGD motifs were presented in a biologically relevant manner. Therefore, the derived dissociation constant values inherently reflect the effective, functional fraction of the immobilized S1 protein. See Table 2.

To minimize nonspecific adhesion, PP blocking was applied to surfaces coated with 0.267 µM S1 protein (Figure 2B). Despite the blocking treatment, HeLa cells adhered robustly to the S1-coated surface, exhibiting a characteristic sigmoid response curve. In contrast, surfaces treated only with PP or left uncoated showed minimal adhesion, indicating that the observed signal was specifically driven by integrin–S1 interactions. These findings demonstrate that the RGD-like motif within the immobilized S1 protein remains accessible and functionally competent, even in the presence of anti-fouling treatment. To further verify the specificity of this interaction, soluble S1 protein was added to the cell suspension during adhesion to a 50% PPR-coated surface (Figure 2C). The presence of soluble S1 significantly reduced cell attachment compared to controls without S1 or with BSA in solution, suggesting competitive inhibition at the level of integrin binding. This supports the hypothesis that SARS-CoV-2 S1 directly targets RGD-recognizing integrins.

To investigate how different concentrations of surface-immobilized S1 protein affect cell adhesion, a dilution series of S1 protein (ranging from 0.085 to 0.267 µM) was applied to the biosensor surface, followed by PP blocking and the addition of HeLa cells (Figure 2D). The real-time adhesion curves revealed a dose-dependent increase in Δλ, which reached a clear plateau at the highest S1 concentration, indicating saturation of available integrin-binding sites. Notably, the response observed at 0.267 µM was significantly higher than that at lower concentrations, suggesting a cooperative effect or threshold concentration required for optimal surface presentation of the RGD motifs. The similarity in adhesion kinetics between the S1-coated and the 50% PPR-coated surfaces indicates that the immobilized S1 retained sufficient biological activity for integrin binding. To rule out multilayer adsorption, the calculated surface mass density (116 ng/cm^2^) and protein spacing confirmed the formation of a compact monolayer, which remained biologically active based on continued high adhesion at saturation. This sharp transition between sub-saturating and saturating concentrations could also reflect the nonlinear relationship between S1 density and the availability of integrin-accessible epitopes on the immobilized protein layer.

In the next experiment, we assessed the competitive inhibitory effect of soluble S1 on integrin-mediated adhesion. For this, increasing concentrations of soluble S1 were added simultaneously with HeLa cells onto a surface coated with 50% PPR (Figure 2E). A clear inverse relationship was observed between S1 concentration and Δλ_max_ values, confirming that soluble S1 competes effectively with surface-bound RGD motifs for integrin binding. This finding also validates the plateau concentration assumed in Figure 2C, indicating that the S1 concentration used there was sufficient to achieve maximal inhibitory effect.

The normalized adhesion responses were fitted with a sigmoidal curve to estimate the IC_50_ value (Figure 2F). The fitted curve supports the conclusion that soluble S1 blocks integrin engagement in a concentration-dependent manner, further substantiating the specificity and direct nature of the S1–integrin interaction.

### Kinetic Modeling and Prediction of the Dissociation Constant of S1-Integrin Interaction

The dissociation constant is a key parameter that characterizes the affinity between a receptor and its ligand. In our system, it reflects the binding strength between the surface-immobilized SARS-CoV-2 S1 spike protein and RGD-specific integrins expressed by HeLa cells. Lower dissociation constant values indicate stronger interactions and, therefore, higher affinity. This measurement is especially important in cellular systems, where ligand-receptor interactions are influenced not only by biochemical affinity but also by spatial distribution and membrane organization.

Kanyo et al., (2020) developed a kinetic model to interpret adhesion curves measured by the RWG biosensor, incorporating key molecular-level interactions [15]. The model uses a system of coupled ordinary differential equations to describe how the surface concentrations of ligands, free integrins, and integrin–ligand complexes evolve over time. The recruitment of integrins to the adhesion zone is governed by specific kinetic rate constants. Accordingly, the time-dependent changes in the surface concentrations of ligand (L), free integrin (I), and the integrin–ligand complex (B) in the adhesion zone are described by the following equations:

(3) $$\frac{dB}{dt}=k_{1}L\cdot I-k_{2}B$$

(4) $$\frac{dL}{dt}=-k_{1}L\cdot I+k_{2}B$$

(5) $$\frac{dI}{dt}=-k_{1}L\cdot I+k_{2}B+k_{3}B\left(I_{\max}-I\right)$$

In this model, k_1_ and k_2_ denote the integrin–ligand association and dissociation rate constants, respectively, while k_3_ represents the rate at which integrins are recruited into the adhesion zone. Iₘₐₓ defines the upper limit of free integrin density in that zone; once the free integrin concentration I reaches Iₘₐₓ, no further receptors can be recruited. The sensor’s measured wavelength shift is proportional to the surface density of integrin–ligand complexes (B) formed during adhesion.

To match the experimental data with the kinetic model, it was necessary to convert the wavelength shift signals, recorded in picometers (pm), into surface concentrations expressed as integrins per square micrometer. Calibration was based on findings showing that a maximum wavelength shift of approximately 1200 pm corresponds to full saturation of integrin binding in the adhesion zone beneath HeLa cells. According to Deschout et al., (2014) [27], PALM-based super-resolution microscopy revealed that typical adherent mammalian cells engage approximately 6000 integrins with the substrate, distributed across a contact area of ~1000 μm^2^. Based on this, a direct proportionality was established: a shift of 1200 pm corresponds to an average surface concentration of 6 integrins/μm^2^, yielding a conversion factor of 1 pm ≈ 0.005 integrins/μm [2,27]. This proportional relationship forms the basis of the calibration and allows experimental wavelength shift data to be translated into integrin surface concentrations for model fitting [2,15].

**Figure 3:**
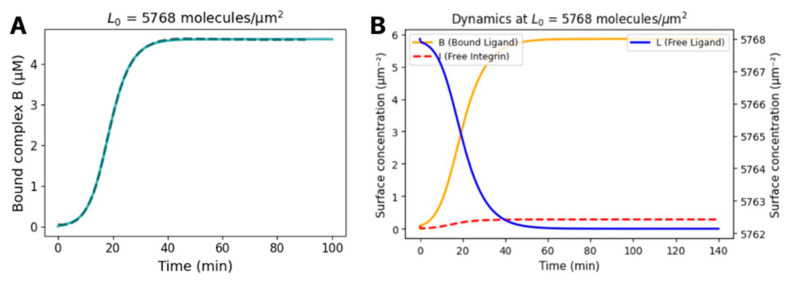
Adhesion kinetics and model fitting of integrin–S1 interaction in live cells. (**A**) Time course of bound integrin–ligand complex concentration upon seeding HeLa cells onto S1-coated surfaces (L_0_ = 5768 molecules·µm^−2^, corresponding to 0.267 µM S1 surface concentration). Data represent *n* = 5 technical replicates. Gray dashed line: experimental Δλ-derived concentration. Teal solid line: fit of the kinetic model yielding rate constants *k*_1_, *k*_2_, *k*_3_ and maximum complex density *Iₘₐₓ*. *n* = 5. (**B**) Simulated dynamics of complex (B, orange solid), free-integrin (red dashed), and free-ligand (blue solid) surface concentrations over 140 min using the fitted rate constants.

The two-dimensional dissociation constant ($K_{d}^{S1}$) we determined and converted into a conventional three-dimensional KdS13D  value by dividing by the confinement height (100 nm) and Avogadro’s number (6.022 × 10^23^ mol^−1^). This calculation gives a volumetric KdS1 3D value of 4.62 ± 0.03 µM, allowing us to directly compare our surface-based measurements with typical solution-phase binding affinities. Integrin–ligand interactions are known to be cooperative and dynamic, often involving receptor clustering and cytoskeletal recruitment. Therefore, a kinetic model that incorporates time-resolved data and receptor mobility offers a more appropriate description of these processes [28]. This approach also helps to explain the moderate steepness of the initial adhesion curve (Figure 3A), which likely reflects biologically relevant weak cooperativity, such as localized integrin clustering or activation. Although the kinetic model captures this general behavior, it may slightly overestimate cooperativity due to simplifications [29]. The 100 nm value used in this calculation corresponds to the effective thickness of the cell–surface confinement or binding zone. This is consistent with the confinement zone thickness concept described by Kanyo et al. in 2020 [15], where an estimated thickness of ~60–100 nm was used to bridge two-dimensional (surface-bound) and three-dimensional (solution-phase) affinity models. Specifically, this thickness represents the average nanoscale distance between the cell membrane and the functionalized substrate surface where integrin–ligand interactions physically occur.

As shown in Figure 3A, the time course of bound integrin–ligand complex formation closely follows the kinetic model fit (teal solid line), validating the extracted rate constants. Parallel time courses supporting Figure 3A are shown in Figure S1. Figure 3B illustrates the simulated dynamics of bound complex (B), free-integrin (I), and free-ligand (L) surface concentrations, highlighting the rapid initial binding and subsequent approach to steady state.

To further validate the KdS1 3D obtained from our surface-based kinetic model, we conducted an independent inhibition assay using soluble S1 protein, following a strategy similar to that used by Székács et al. (2018) [16]. In this setup, HeLa cells were seeded onto a 50% PPR-coated surface, while increasing concentrations of S1 protein were added in solution. The normalized adhesion response was fitted with a sigmoidal curve (Figure 2F) yielding an IC_50_ of 1.4 ± 0.40 µM.

Using the method described in Szekacs et al., the corresponding KdS1 3D value can be calculated as [16]:

(6) KdS1   3D=IC501+[RGD]KdRGD  

where [RGD] is the estimated concentration of surface-bound RGD ligands, and $K_{d}^{RGD}$ is the dissociation constant of the surface interaction. In our case, [RGD] was estimated to be 2.39 µM, and the fitted KdRGD 3D was 9.4 ± 0.97 µM, as determined from a new batch of PPR. To obtain this value, HeLa cells were seeded on surfaces coated with increasing concentrations of the new RGD batch, and adhesion responses were recorded using an RWG-based optical biosensor (Figure S2).

Substituting these values into the equation, we obtained a calculated KdS1 3D value of 1.116 µM. Of note, this value is consistent with the predictions of Ref. [11]. To interpret this value in the context of S1 protein functionality, we simulated how different levels of S1 activity would influence the calculated KdS1 3D (Figure S3). The fitted linear model indicated that a KdS1 3D value of 1.116 µM corresponds to approximately 33% of the immobilized S1 protein is functionally active and available to cellular integrins. This suggests that under our experimental conditions, about one-third of the surface-bound S1 retained an integrin-accessible conformation capable of mediating specific binding. This also confirms that the assumption of full activity would significantly underestimate the true dissociation constant. Therefore, our results should be interpreted as reflecting the effective binding affinity corresponding to the functional protein fraction.

Several integrin subtypes are present on the HeLa surface and the S1 subunit is displayed in a random orientation relative to these receptors. The measured Kd is directly comparable to the value we previously reported in Orgovan et al. [2], where we used the same RWG method to quantify the interaction between RGD motifs and integrins of HeLa cells. Interestingly, when the receptor was studied in its isolated, non–membrane-bound form, the affinity differed markedly [9,10,11]. Although the full S-protein contains an RGD sequence, structural analyses suggest that it is not presented in an ideal conformation for integrin binding [26,30]; thus, its affinity remains limited compared to engineered systems where the RGD motif is optimally exposed.

## 4. Conclusions

In this study, we quantitatively characterized the interaction between the full-length SARS-CoV-2 S1 subunit and RGD-specific integrins on ACE2-negative HeLa cells, employing a high-throughput RWG biosensor platform. We quantified real-time adhesion kinetics and derived both 2D and 3D dissociation constants of the integrin–S1 interaction. The calculated dissociation constant values (~4.6 µM from kinetic modeling and 1.1 µM from inhibition assays) indicate moderate affinity, consistent with partial integrin accessibility and in a reasonable agreement with the predictions of prior SPR data [11]. Our results confirm that the immobilized S1 protein forms a compact monolayer, and that its RGD motif retains biological activity; however, only a fraction of the immobilized molecules (~33%) remain functionally accessible to integrins. This indicates that the presentation of the RGD motif is suboptimal compared to synthetic peptides, likely due to random orientation or partial conformational masking upon adsorption. These findings support integrin–S1 binding as a plausible auxiliary entry route for SARS-CoV-2 and highlight the potential of biosensor-based approaches for dissecting virus–host interactions with high spatiotemporal resolution.

## Figures and Tables

**Table 1:** Published and present binding affinities between the SARS-CoV-2 S1 protein and various integrin subtypes, with corresponding methods and reference studies.

Integrin Target	Viral Ligand	Reported KdS13D(nM)	Method	Reference Study
αVβ6	recombinant SARS-CoV-2 S1-RBD (RGD-containing)	230 ± 180	SPR	Norris, 2022 [11]
α5β1	recombinant full S1 domain (not just the RBD, but the larger S1 region of the spike protein) (biotin-S1)	31 ± n.d.	SPR (1:1 kinetic fit)	Liu, 2022 [9]
αVβ3	recombinant SARS-CoV-2 S1-RBD peptide (RGD-containing)	>500	SPR	Norris, 2022 [11]
αVβ1	recombinant RGD-peptide from S-protein	50–100	In vitro integrin-binding assay	Bugatti, 2022 [10]
α5β1, αvβ5, αvβ3	recombinant human coronavirus SARS-CoV-2 Spike Glycoprotein S1 (Active)	4616 ± 252 and 1116 ± 0.040	RWG biosensor-based whole cell interaction assay (from adhesion kinetics and competitive adhesion assay)	present study

**Table 2:** Calculated surface parameters from optical biosensor measurements across serial dilutions of S1 protein.

Concentration (µM)	Δλ_corr (pm)	Γ (ng/cm^2^)	$\rho_{RGD}$(nmol/m^2^)	d_RGD-RGD_ (nm)	ν_RGD_ (μm^−2^)
2.67 × 10^−1^	1003.10	116	9.28 × 10^−4^	14.40	5768
5.33 × 10^−2^	315.64	23.2	1.85 × 10^−4^	32.1	1113
1.06 × 10^−2^	240.55	4.64	3.71 × 10^−5^	71.9	223
2.13 × 10^−3^;	249.78	0.928	7.42 × 10^−6^	160.7	44.5
4.27 × 10^−4^	233.64	0.19	1.48 × 10^−6^	359.4	8.9
8.53 × 10^−5^	64.98	0.03712	2.97 × 10^−7^	803.6	2

## Data Availability

The data used to support the findings of this study are available from the corresponding author upon request.

## Supplementary Materials

The following supporting information can be downloaded at: https://www.mdpi.com/article/10.3390/bios15080534/s1, Figure S1. Time-dependent formation of integrin–ligand complexes upon HeLa cell adhesion to S1-coated surfaces: HeLa cells were seeded onto surfaces coated with 0.267 µM SARS-CoV-2 S1 protein, corresponding to a ligand surface density of 5768 molecules/µm^2^. Each panel (A–F) represents an independent technical replicate (n = 5), showing the time course of integrin–ligand complex formation (in µM) derived from real-time biosensor signals. Gray dashed lines indicate experimental Δλ-based concentrations, while solid blue lines show fitted curves from the kinetic adhesion model. The close agreement between replicates confirms the reproducibility of the kinetic response under saturating S1 coating conditions. Figure S2. Determination of the dissociation constant $K_{D}^{RGD}$ for the newly used RGD peptide batch in surface functionalization: Maximum wavelength shifts (Δλ_max_) measured during HeLa cell adhesion were plotted as a function of RGD surface density (ν_RGD_, molecules/µm^2^). The data were fitted with a sigmoidal binding curve to determine the apparent two-dimensional dissociation constant: $K_{d}^{RGD}$ = 563.96 ± 58.23 molecules/µm^2^. This value was converted into a three-dimensional dissociation constant: 3D $K_{d}^{RGD}$ = 9.40 ± 0.97 µM. The fitted curve reveals the saturation behavior of integrin binding in response to increasing ligand density. Figure S3. Effect of S1 protein activity on the calculated 3D dissociation constant (3D $K_{D}^{S1}$). To assess how the functional activity of the immobilized S1 protein affects integrin binding affinity, we performed a simulation linking surface activity levels to the apparent 3D dissociation constant (3D $K_{D}^{S1}$ The simulation was based on sorted experimental values and assigned activity levels from 5% to 100%. Local neighborhood averaging was used to smooth the data, and the results were plotted on a semi-logarithmic scale. The fitted curve captures the expected trend: as S1 surface activity increases, the apparent 3D $K_{D}^{S1}$ decreases, indicating stronger integrin binding. A 3D $K_{D}^{S1}$ value of 1.116 µM—measured from competitive adhesion experiments with soluble S1—corresponds to approximately 33% functional activity, suggesting that only one-third of the immobilized S1 remains capable of engaging integrins. Figure S4. Representative contact angle measurements on Nb_2_O_5_-coated biosensor surfaces to assess surface wettability. Static water droplets were analyzed using low-bond axisymmetric drop shape analysis. The measured contact angles ranged from 61.8° to 76.3°, indicating intermediate wettability, which is favorable for preserving protein structure upon adsorption. Among the tested drops, the image shown in panel 47 (contact angle ≈ 76.3°) represents the highest observed contact angle, while panel 46 and 45 (≈62–63°) correspond to the lower end of the range, confirming consistency across replicate surfaces. Panel 41 represents a smaller drop with reduced volume (0.0604 mm^3^), still falling within the expected wettability range. These findings support the suitability of the Nb_2_O_5_ surface for biosensor assays.
