# Correction: Kanyo et al. Kinetic Analysis of SARS-CoV-2 S1–Integrin Binding Using Live-Cell, Label-Free Optical Biosensing. *Biosensors* 2025, *15*, 534

**DOI:** 10.3390/bios16020068

**Published:** 2026-01-23

**Authors:** Nicolett Kanyo, Krisztina Borbely, Beatrix Peter, Kinga Dora Kovacs, Anna Balogh, Beatrix Magyaródi, Sandor Kurunczi, Inna Szekacs, Robert Horvath

**Affiliations:** 1 Nanobiosensorics Laboratory, Institute of Technical Physics and Materials Science, HUN-REN Centre for Energy Research, Konkoly-Thege Miklós út 29-33, H-1121 Budapest, Hungary; kanyo.nicolett@ek.hun-ren.hu (N.K.); borbely.krisztina@ek.hun-ren.hu (K.B.); peter.beatrix@ek.hun-ren.hu (B.P.); kovacs.kinga.dora@ek.hun-ren.hu (K.D.K.); balogh.anna@ek.hun-ren.hu (A.B.); magyarodi.beatrix@ek.hun-ren.hu (B.M.); kurunczi.sandor@ek.hun-ren.hu (S.K.); inna.szekacs@ek.hun-ren.hu (I.S.); 2 Chemical Engineering and Material Science Doctoral School, University of Pannonia, Egyetem u.10, H-8200 Veszprém, Hungary; 3 Department of Biological Physics, Eötvös University, Pázmány Péter Sétny. 1/C, H-1117 Budapest, Hungary; 4 Institute of Biophysics, HUN-REN Biological Research Centre, H-6726 Szeged, Hungary

## Text Correction

Some corrections have been made to the original publication [1]. The revised version is as follows:

The kinetic dissociation constant (KdS13D) was previously reported as 4.616 ± 0.252 µM, but should read 12.76 ± 6.916 µM.

The statement “L_0_ = 5768 molecules·µm^−2^, corresponding to 0.267 µM S1 surface concentration” should read “L_0_ = 5768 molecules·µm^−2^, derived from a 0.267 µM coating solution.”

In the Supplementary Information, the sentence “The fitted curve captures the expected trend: as S1 surface activity increases, the apparent 3D $K_{D}^{S1}$ decreases, indicating stronger integrin binding” should read “The fitted curve captures the expected trend: as S1 surface activity increases, the apparent 3D $K_{D}^{S1}$ increases, indicating weaker integrin binding at higher apparent S1 activities.”

## Error in Figure

The *y* axis units in Figure 3 and the Supplementary Information were incorrectly labeled in µM; they should be in molecules·µm^−2^. Corrected versions of Figure 3 and Figure S1 are presented below. We also changed text in the figure caption for clarity.

**Figure 3:**
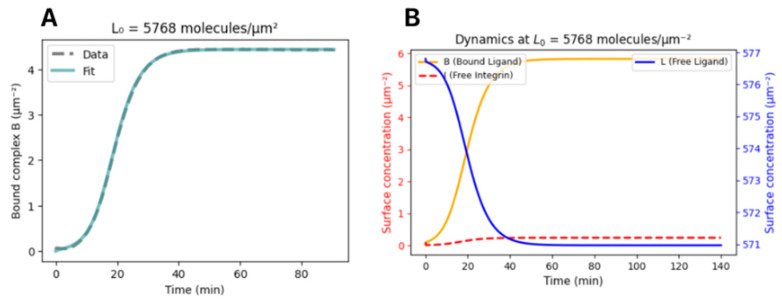
Adhesion kinetics and model fitting of integrin–S1 interaction in live cells. (**A**) Time course of bound integrin–ligand complex concentration upon seeding HeLa cells onto S1-coated surfaces (L_0_ = 5768 molecules·µm^−2^, derived from a 0.267 µM coating solution). Data represent *n* = 5 technical replicates. Gray dashed line: Bound complex surface concentration calculated from the experimental Δλ change using the calibration equation. Teal solid line: Fit of the kinetic model yielding rate constants *k*_1_, *k*_2_, and *k*_3_ and maximum complex density *I_max_*. *n* = 5. (**B**) Simulated dynamics of complex (B, orange solid), free-integrin (red dashed), and free-ligand (blue solid) surface concentrations over 140 min using the fitted rate constants.

**Figure S1:**
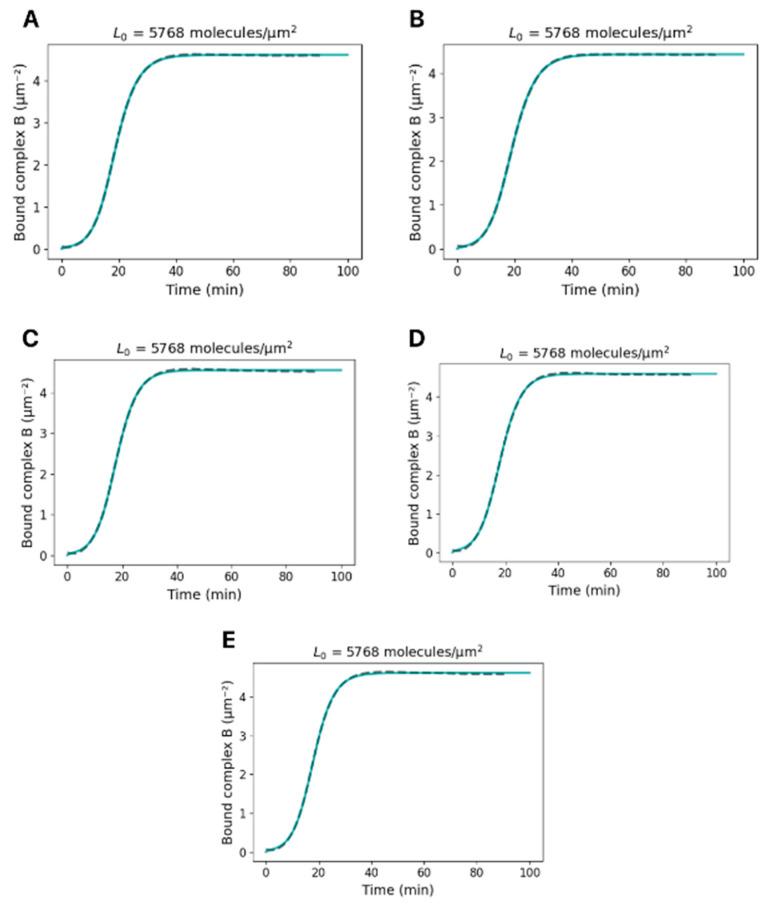
**Time-dependent formation of integrin–ligand complexes upon HeLa cell adhesion to S1-coated surfaces:** HeLa cells were seeded onto surfaces coated with 0.267 µM SARS-CoV-2 S1 protein, corresponding to a ligand surface density of 5768 molecules/µm^2^. Each panel (**A**–**E**) represents an independent technical replicate (*n* = 5), showing the time course of integrin–ligand complex formation (in µm^−2^) derived from real-time biosensor signals. Gray dashed lines indicate experimental Δλ-based concentrations, while solid blue lines show fitted curves from the kinetic adhesion model. The close agreement between replicates confirms the reproducibility of the kinetic response under saturating S1 coating conditions.

This correction was approved by the Academic Editor. The original publication has also been updated.
