# Supplementary material for: Kinetic Analysis of SARS-CoV-2 S1–Integrin Binding Using Live-Cell, Label-Free Optical Biosensing
Source: Biosensors (Basel). 2025 Aug 14;15(8):534. doi: 10.3390/bios15080534 (PMC12384205; doi:10.3390/bios15080534)
Supplement: Supplementary file 1 [file biosensors-15-00534-s001.zip › biosensors-3706066-supplementary.pdf]

biosensors

MDPI

supplementary

Kinetic Analysis of SARS-CoV-2 S1–Integrin Binding Using Live-Cell, Label-Free Optical Biosensing

Nicolett Kanyo [1,2], Krisztina Borbely [1,2], Beatrix Peter [1], Kinga Dora Kovacs [1,3], Anna Balogh [1,3], Beatrix Magyaródi [1,2], Sandor Kurunczi [1], Inna Szekacs [1] and Robert Horvath [1,4,*]

[1] Nanobiosensorics Laboratory, Institute of Technical Physics and Materials Science, HUN-REN Centre for Energy Research, Konkoly-Thege Miklós út 29-33, H-1121 Budapest, Hungary; kanyo.nicolett@ek.hun-ren.hu (N.K.); borbely.krisztina@ek.hun-ren.hu (K.B.); peter.beatrix@ek.hun-ren.hu (B.P.); kovacs.kinga.dora@ek.hun-ren.hu (K.D.K.); balogh.anna@ek.hun-ren.hu (A.B.); magyarodi.beatrix@ ek.hun-ren.hu (B.M.); kurunczi.sandor@ek.hun-ren.hu (S.K.); inna.szekacs@ek.hun-ren.hu (I.S.)

[2] Chemical Engineering and Material Science Doctoral School, University of Pannonia, Egyetem u.10, H-8200 Veszprém, Hungary

[3] Department of Biological Physics, Eötvös University, Pázmány Péter Sétny. 1/C, H-1117 Budapest, Hungary

[4] Institute of Biophysics, HUN-REN Biological Research Centre, H-6726 Szeged, Hungary

* Correspondence: horvath.robert@ek.hun-ren.hu

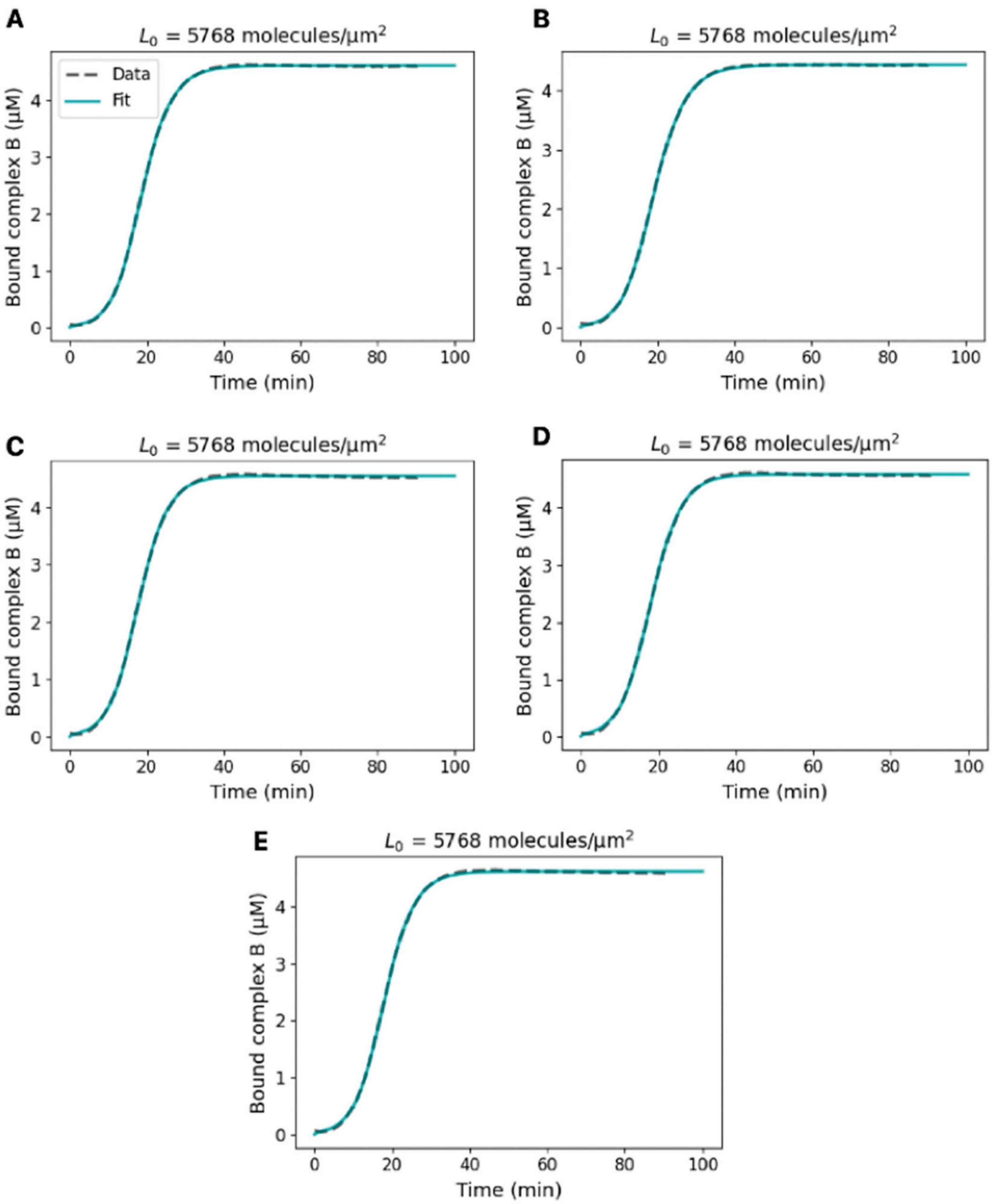

Figure S1. Time-dependent formation of integrin–ligand complexes upon HeLa cell adhesion to S1-coated surfaces: HeLa cells were seeded onto surfaces coated with 0.267 µM SARS-CoV-2 S1 protein, corresponding to a ligand surface density of 5768 molecules/µm². Each panel (A–F) represents an independent technical replicate (n = 5), showing the time course of integrin–ligand complex formation (in µM) derived from real-time biosensor signals. Gray dashed lines indicate experimental Δλ-based concentrations, while solid blue lines show fitted curves from the kinetic adhesion model. The close agreement between replicates confirms the reproducibility of the kinetic response under saturating S1 coating conditions.

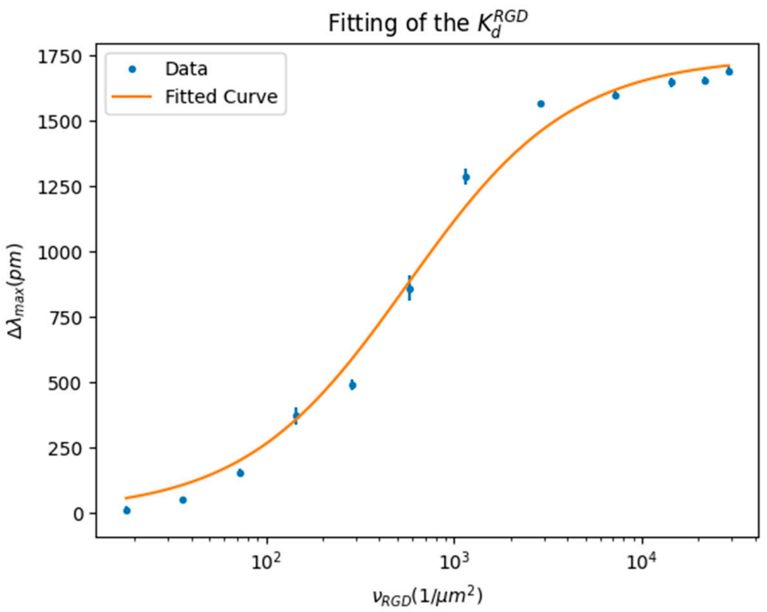

Figure S2. Determination of the dissociation constant K_D^{RGD} for the newly used RGD peptide batch in surface functionalization: Maximum wavelength shifts ($\Delta\lambda_{max}$) measured during HeLa cell adhesion were plotted as a function of RGD surface density (ν_{RGD}, molecules/μm²). The data were fitted with a sigmoidal binding curve to determine the apparent two-dimensional dissociation constant: K_d^{RGD} = 563.96 ± 58.23 molecules/μm². This value was converted into a three-dimensional dissociation constant: 3D K_d^{RGD} = 9.40 ± 0.97 μM. The fitted curve reveals the saturation behavior of integrin binding in response to increasing ligand density.

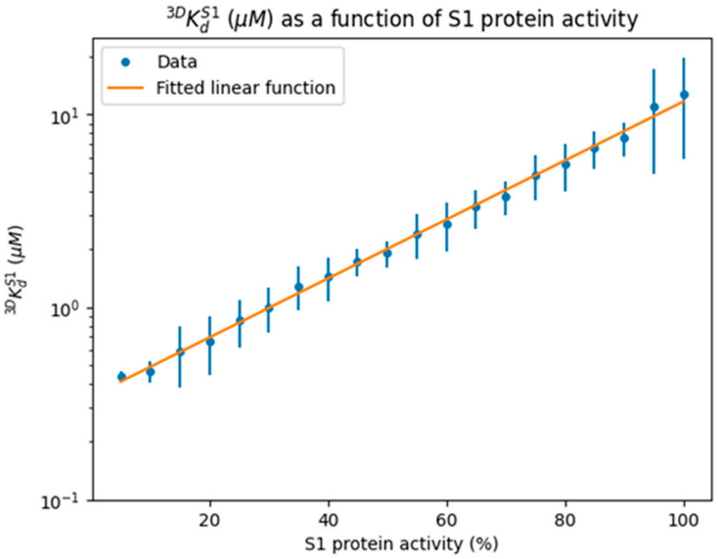

Figure S3. Effect of S1 protein activity on the calculated 3D dissociation constant (3D K_D^{S1}). To assess how the functional activity of the immobilized S1 protein affects integrin binding affinity, we performed a simulation linking surface activity levels to the apparent 3D dissociation constant (3D K_D^{S1}The simulation was based on sorted experimental values and assigned activity levels from 5% to 100%. Local neighborhood averaging was used to smooth the data, and the results were plotted on a semi-logarithmic scale. The fitted curve captures the expected trend: as S1 surface activity increases, the apparent 3D K_D^{S1}decreases, indicating stronger integrin binding. A 3D K_D^{S1}value of 1.116 μM—measured from competitive adhesion experiments with soluble S1—corresponds to approximately 33% functional activity, suggesting that only one-third of the immobilized S1 remains capable of engaging integrins.

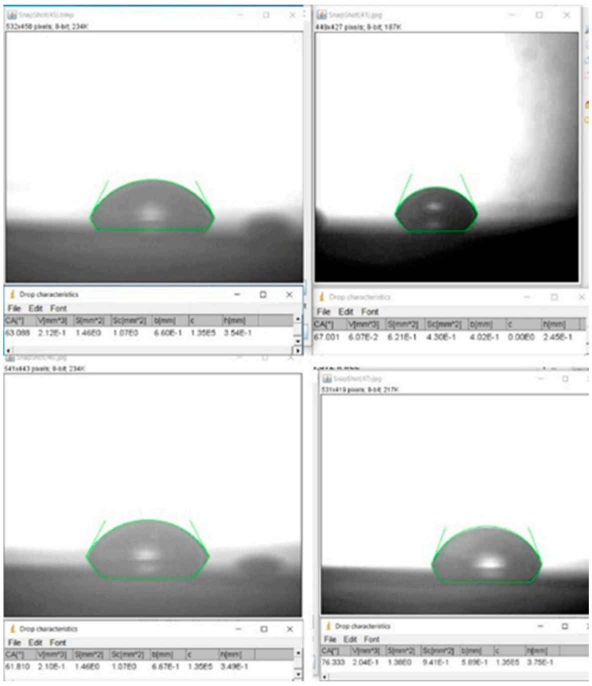

Figure S4. Representative contact angle measurements on Nb_2O_5-coated biosensor surfaces to assess surface wettability. Static water droplets were analyzed using low-bond axisymmetric drop shape analysis. The measured contact angles ranged from 61.8° to 76.3°, indicating intermediate wettability, which is favorable for preserving protein structure upon adsorption. Among the tested drops, the image shown in panel 47 (contact angle ≈ 76.3°) represents the highest observed contact angle, while panel 46 and 45 (≈62–63°) correspond to the lower end of the range, confirming consistency across replicate surfaces. Panel 41 represents a smaller drop with reduced volume (0.0604 mm^3), still falling within the expected wettability range. These findings support the suitability of the Nb_2O_5 surface for biosensor assays.
